# Loss of Pol32, a subunit of DNA polymerases δ and ζ, leads to different patterns of genome stability than direct impairment of these individual polymerases

**DOI:** 10.1128/mbio.00531-26

**Published:** 2026-04-17

**Authors:** Lei Qi, Ke-Jing Li, Xing-Xing Tang, Li-Yan Tian, Ye-Ke Wang, Min He, Ke Zhang, Thomas D. Petes, Dao-Qiong Zheng

**Affiliations:** 1 State Key Laboratory (SKL) of Biobased Transportation Fuel Technology, Ocean College, Zhejiang University, Zhoushan, China; 2 Department of Molecular Genetics and Microbiology, Duke University, Durham, North Carolina, USA; 3 College of Life Science, Zhejiang University 12377, Hangzhou, China; Instituto Carlos Chagas, Curitiba, Brazil

**Keywords:** Pol32, genome integrity, DNA polymerases, Rev3, mitotic recombination

## Abstract

Pol32 is a subunit shared by DNA polymerases δ and ζ, yet its role in maintaining genome integrity remains incompletely defined. Here, we employed whole-genome sequencing of mutation-accumulation lines to systematically characterize the genome-wide effects of a *POL32* deletion in diploid *Saccharomyces cerevisiae*. Loss of Pol32 led to substantially (>5-fold) elevated rates of loss of heterozygosity (LOH), chromosome rearrangements, and aneuploidy, but resulted in substantially less genome instability than observed in strains with low levels of DNA polymerase δ. In particular, there was only a small (<2-fold) effect of the *pol32* mutation on mutation rates. Notably, a prominent hotspot for chromosome rearrangements located near the end of chromosome VII was observed in *pol32* strains. Although deletion of *REV3* (encoding the catalytic subunit of Pol ζ) had no significant effect on genome integrity in a wild-type background, *pol32 rev3* double mutants had reduced rates of most types of chromosome alterations compared to the *pol32* single mutant, implicating Pol ζ in driving the genome instability induced by the Pol32 deficiency. Together, these findings provide new insights into how a shared structural subunit of several DNA polymerases contributes to the regulation of genome stability.

**IMPORTANCE:**

Pol32 is a subunit of DNA polymerases δ (an essential replicative enzyme) and ζ (an error-prone DNA polymerase required for DNA repair). We show that yeast strains that lack this protein have elevated rates of mitotic recombination, large deletions/duplications, translocations, and other types of genomic alterations. The high level of genomic alterations in *pol32* mutants is substantially suppressed in strains that lack DNA polymerase ζ, suggesting that this error-prone polymerase may stimulate DNA breaks in conditions of DNA replication stress. Our studies are likely to have wide relevance since sequence variants of POLD3 (the human homolog of Pol32) are associated with certain types of human tumors.

## INTRODUCTION

In eukaryotic cells, genome replication requires a tight coordination of the three high-fidelity replicative DNA polymerases (Pols) α, ε, and δ. Leading strand synthesis is mainly carried out by Pol ε, while on the lagging strand, Pol α synthesizes the RNA primer and a short stretch of DNA, which is extended by Pol δ (1). DNA damage repair also relies on error-prone polymerases (2, 3). For example, Pol ζ plays a role in translesion synthesis (TLS) that allows the replication machinery to bypass lesions or distortions in the DNA template that would otherwise stall DNA polymerases ε and δ (4, 5).

The polymerases described above contain a catalytic subunit and one or more accessory subunits. For instance, *Saccharomyces cerevisiae* Pol δ consists of Pol3 (catalytic subunit), Pol31, and Pol32 (6). Pol32 also functions as an accessory subunit for Pol ζ in both yeast and human (4, 5, 7–9). Although *POL32* is non-essential in yeast, its deletion becomes lethal in the presence of certain viable point mutations in the essential gene *POL31* (10, 11).

Disruption of Pol32 function in *S. cerevisiae* results in heightened sensitivity to agents that impede replication fork progression, such as methyl methanesulfonate (MMS) or hydroxyurea (HU) (11, 12). Using gene-specific assays, Gerik et al. found that *pol32* strains had a weak (1.6- to 2-fold) antimutator phenotype (6). In addition, using systems to select loss of heterozygosity (LOH), Yuen et al. and Andersen et al. showed that *pol32* strains had elevated rates of chromosome loss and mitotic recombination for the marked chromosomes (13, 14). Furthermore, Pol32-deficient cells are impaired in break-induced replication (BIR) pathway (15). The loss of Pol32 also leads to reduced Pol3 and Pol31 protein levels (11), and *pol32* strains exhibit a delay at the G2/M phase of the yeast cell cycle (16). In humans, germline mutations or common variations in POLD3 (encoding the Pol32 human homolog) predispose to hypermutation and cancers (17). However, it remains unclear how a deficiency in Pol32/POLD3 function affects the full spectrum of genomic alterations, particularly at the whole-genome level.

In *S. cerevisiae*, our previous studies showed that reduced expression of the catalytic subunits of Pols α, ε, and δ greatly stimulates global genome instability, resulting in higher rates of aneuploidy, chromosomal rearrangements, LOH, and point mutations (18–20). Reduction in the level of different replicative DNA polymerases can result in different patterns of genomic alterations. For example, cells with reduced Pol2 levels (the catalytic subunit of Pol ε) exhibit shorter telomeres and accumulate LOH events in telomeric regions (19), phenotypes that are not observed in cells with reduced Pol3 or Pol1 (18, 20).

These findings highlight the division of labor among different polymerases in maintaining genome integrity. In comparison to a reduction in the level of catalytic subunits of DNA polymerases, it remains to be determined whether deficiencies in accessory subunits, such as Pol32, result in similar or distinct patterns of genomic alterations. In this study, we investigated the global patterns of genomic alterations in Pol32-deleted yeast strains using mutation-accumulation experiments and whole-genome sequencing. We identified unique signatures of genetic variations in the *pol32* mutant, including changes in the mutation spectrum and other genome rearrangements. These findings offer insights into how loss of Pol32 influences genome stability.

## RESULTS

Below, we first describe how loss of Pol32 leads to elevated rates of mitotic recombination, large deletions and duplications, aneuploidy, and other genomic changes. Second, we will examine the genetic interactions that occur between the *pol32* and *rev3* (the gene encoding the error-prone DNA polymerase ζ).

### Loss of heterozygosity (LOH) events detected throughout the genome in *pol32* diploids by mutation-accumulation analysis

This study used a diploid (WYpol32, Tables S1 and S2) that was homozygous for the *pol32* deletion. The diploid was generated by crossing two haploid strains that differed by about 50,000 SNPs, allowing high-resolution mapping of various types of chromosome alterations (21–23). We propagated 46 isolates of WYpol32 from a single cell to a colony on rich medium (YPD) at 30°C to allow accumulation of genetic variants. Thirty-eight isolates underwent 8 generations of sub-culturing, whereas 8 were sub-cultured for 20 generations. These isolates were then analyzed using whole-genome sequencing with a 2 × 150 bp paired-end strategy. Additionally, eight isolates were re-sequenced using long-read technology (Oxford Nanopore). Based on the total number of events in each category, the number of isolates, the number of cycles of sub-cloning per isolate, and the number of doublings required to form a colony, we calculated the rate of alterations per cell division (details in Supplemental Information); the criteria that define each genomic alteration are also described in Supplemental Information.

**Fig 1:**
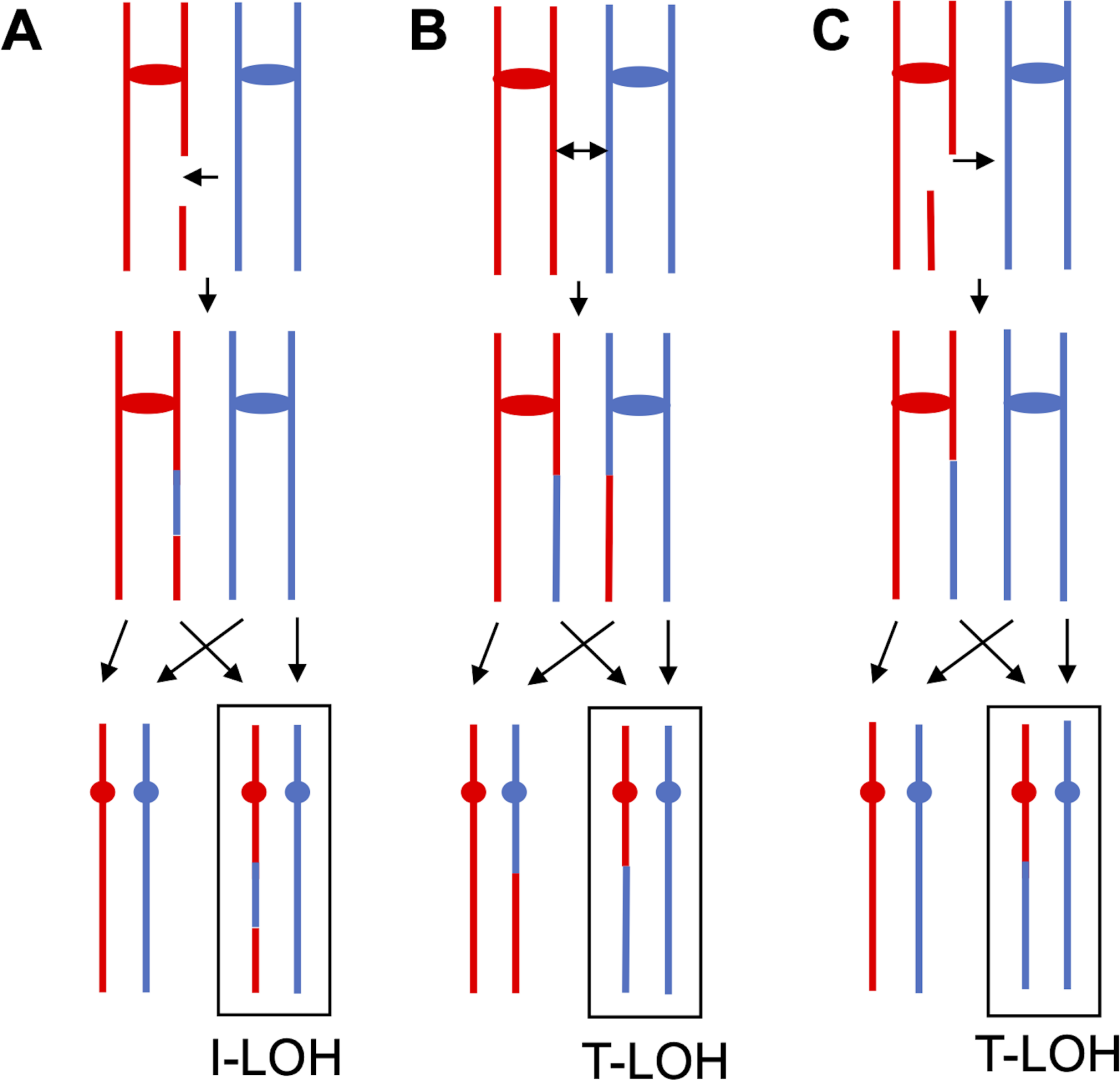
Common patterns of mitotic recombination in diploid strains heterozygous for densely-located SNPs. The chromosomes are shown following DNA replication but before chromosome segregation. Horizontal arrows indicate the position of double-strand DNA breaks (DSBs) that initiate the event. The red and blue colors indicate the two homologs. Although we show the chromosomes of both daughter cells that contain the recombinant products, in sub-cultured isolates, we recover only one of the two products (enclosed in a rectangle). (**A**) Interstitial LOH (I-LOH)/gene conversion unassociated with a crossover. (**B**) Terminal LOH event (T-LOH) resulting from a reciprocal crossover, unassociated with a gene conversion. (**C**) T-LOH event that is the result of break-induced replication. In this pathway, one fragment resulting from the DSB is lost.

In yeast, most LOH events result from repair of DSBs by homologous recombination (HR) (24). Repair of a DSB without an associated crossover (Fig. 1A) will result in interstitial LOH (I-LOH). A mitotic crossover event can result in terminal LOH (T-LOH) in both daughter cells (Fig. 1B). A T-LOH event can also be generated by repair of a DSB during which the terminal fragment of the chromosome is lost (Fig. 1C), a BIR event (25). The chromosome coordinates of breakpoints of 158 I-LOH and 193 T-LOH events detected among the 46 isolates are listed in Data set S1-1. The rates of I-LOH and T-LOH were 5- and 14-fold higher than the rates in isogenic wild-type yeast cells (Table 1). The proportions of I-LOH and T-LOH among all LOH events were 45% (158/351) and 55% (193/351), respectively. These values are different from the wild-type strain (*P* <0.0001, chi-square test) (23), 71% (859/1215) for I-LOH and 29% for T-LOH (356/1215). Possible reasons for this difference will be examined in the Discussion.

**TABLE 1:** Numbers and rates of genome alterations in sub-cultured isolates of a *pol32* (WYpol32) strain compared to a wild-type strain (WYspo11)

Genome alteration^*a*^	Number of events	Rate^*b*^ in *pol32* (×10^−3^ per cell division)	Rate in WT^*c*^ (×10^−3^ per cell division)	Fold of *pol32* rate to wild-type rate^*d*^	Fisher test *P* value^*e*^
I-LOH	158	15* (12–17)^*f*^	3.3 (3.0–3.5)	5	<0.001
T-LOH	193	18* (15–20)	1.3 (1.2–1.5)	14	<0.001
I-DEL	23	2.1* (1.3–3.2)	0.1 (0.08–0.16)	21	<0.001
I-DUP	3	0.28* (0.06–0.81)	0.03 (0.02–0.06)	9	0.012
T-DEL	9	0.83* (0.38–1.6)	0.02 (0.01–0.04)	42	<0.001
T-DUP	8	0.74* (0.32–1.5)	0.01 (0.002–0.03)	74	<0.001
Aneuploidy and UPD	32	3.0* (2.0–4.2)	0.06 (0.04–0.10)	50	<0.001
Single base mutations	89	8.2* (6.6–10.1)	4.8 (4.5–5.1)	1.7	<0.001
Small in/dels and complex mutations	14	1.3* (0.7–2.2)	0.27 (0.21–0.34)	4.8	<0.001

^*a*^ The various types of genomic alterations are described in the text and listed in Data set S1. Events described as I-LOH (interstitial LOH) have a region of LOH flanked by heterozygous SNPs. T-LOH (terminal LOH) events have a breakpoint for LOH that extends to the end of the chromosome. For I-DEL and I-DUP, the coverage of SNPs is reduced by 50% or duplicated by 50%, respectively. In T-DEL and T-DUP events, the change in copy number extends to the end of the chromosome. UPD indicates uniparental disomy events. Small in/dels are insertion or deletion mutations that change one or more base pairs, and complex mutations change more than one base within an 8 bp interval.

^*b*^ Rates were calculated by dividing the number of events by 10,858 (the number of cell divisions/isolate during sub-culturing × the number of isolates). Rates are expressed as ×10^−3^ per cell division. Values in brackets indicate 95% confidence intervals. Other details of the rate calculations are discussed in Supplemental Information.

^*c*^ Rates of alterations in the wild-type strain were determined by reference 23.

^*d*^ The rates calculated for the *pol32* strain were divided by the rates for the isogenic wild-type strain.

^*e*^ The *P* values were calculated using Fisher’s exact test in R Studio.

^*f*^ Asterisks indicate that the wild-type rate was not within the 95% confidence limits of the *pol32* rate.

**Fig 2:**
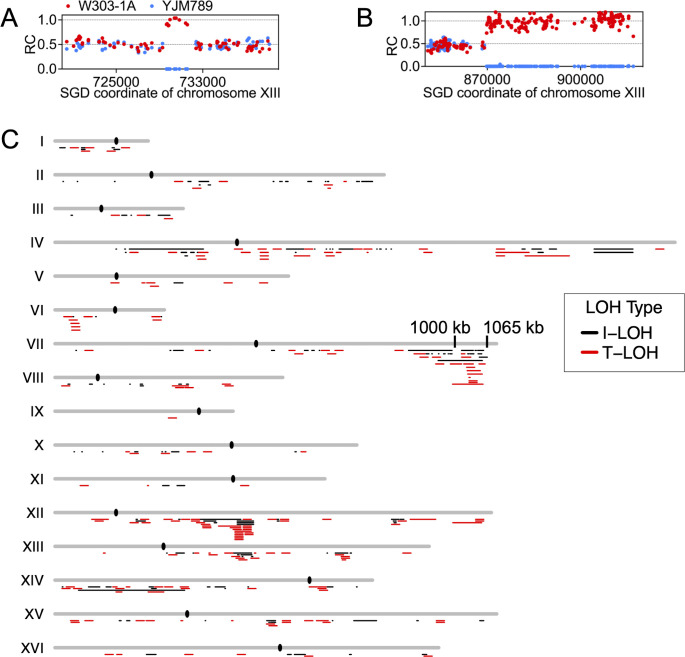
LOH events detected by genomic sequencing of sub-cultured isolates. Each dot shows the relative coverage (RC) for W303-derived (red) and YJM789-derived (blue) SNPs. The RC for each SNP was calculated by dividing the sequencing coverage by the average coverage for all SNPs in the genome. An RC of about 0.5 indicates that the SNP is heterozygous, duplication of a SNP results in an RC of about 1, and deletion of SNPs results in an RC value of 0. The *X*-axis shows the *Saccharomyces* Genome Database for the coordinates on the chromosome. (**A**) Interstitial LOH (I-LOH). I-LOH events result in a chromosome segment in which the SNPs from one homolog are lost and the corresponding set SNPs from the other homolog are duplicated. (**B**) Terminal LOH (T-LOH). T-LOH events involve a single transition between heterozygous and homozygous SNPs with the region of LOH extending to the end of the chromosome. (**C**) Chromosome location of LOH events in sub-cultured isolates. The approximate location of breakpoints for I-LOH (black) and T-LOH (red) events is shown. For I-LOH events, the length of the black lines indicates the distance between the two heterozygous SNPs flanking the homozygous LOH region. For T-LOH events, the breakpoint region is shown as a 20 kb bar that extends 10 kb to each side of the midpoint between the closest heterozygous SNP and the homozygous SNPs; from the breakpoint, the LOH regions extend to the end of the chromosome. Black ovals represent the centromeres.

Simple I-LOH events have two transitions between heterozygous and homozygous SNPs (Fig. 2A), and simple T-LOH events have a single transition (Fig. 2B). In addition, we also observed complex events with multiple transitions, shown schematically in Data set S1-2 (e.g., class c25). In wild-type cells, about 14% (167/1215) of the LOH events are complex (23), whereas *pol32* diploids showed a higher percentage (21%, 74/351; Fisher’s exact test, *P* = 0.001; Data set S1). These complex events are likely generated by a number of different pathways such as “patchy” repair of mismatches in heteroduplexes, formation of symmetric heteroduplexes, and/or template switching during BIR (22, 26).

The higher rates of LOH in *pol32* diploids suggest an increased incidence of recombinogenic DSBs (24). One approach to monitor DSBs is to quantify Rad52 foci, which mark sites of homologous recombination repair (27). Using a fluorescently-tagged Rad52 (details in Supplemental Information), we observed threefold more foci (Fig. S1) in the *pol32* diploid (23 out of 331 cells) than in the isogenic wild-type diploid (7 out of 352 cells); this difference is statistically significant (*P* = 0.002 by Fisher’s exact test). The observation that *pol32* and *rad52* mutations are synthetically lethal (12) is also consistent with an elevated rate of DSBs in *pol32* strains. It should be pointed out that the number of DSBs is not necessarily directly proportional to the frequency of LOH, since a substantial fraction of DSBs are repaired by sister-strand recombination that cannot be detected by LOH (28).

### LOH hotspot on chromosome VII in the *pol32* mutant

The location of LOH events in the *pol32* mutant is shown in Fig. 2C. Although all chromosomes had LOH events, chromosome IX was significantly “cold” (1 out of 351; *P* < 0.001, chi-square test), and there was a significant hotspot near the right end of chromosome VII (22 out of 351; *P* < 0.001, chi-square test). The elevated rate of recombination events in this region could reflect an increased frequency of DSBs or terminal truncations of the right end of chromosome VII.

The hotspot activity on chromosome VII was confirmed by construction of an isogenic *pol32* diploid (QL92) in which the hotspot was flanked by two heterozygous markers, *HIS3* at about 1,000 kb and *URA3* at coordinate 1,065 kb (Fig. S2). A mitotic crossover or BIR event between the centromere and the heterozygous *URA3* marker would generate a Ura^−^ derivative, which is selectable on medium containing 5-fluoroorotate acid (5-FOA) (29). If the breakpoint of the rearrangement was between *HIS3* and *URA3*, the derivative would be 5-FOA^R^ His^+^. A wild-type isogenic diploid (QL90) was constructed with the same insertions. The rate of recombination between *HIS3* and *URA3* in the *pol32* strain was 8.0 × 10^−4^/division, an 85-fold increase compared to the wild-type rate for the same interval of 9.4 × 10^−6^/division; this increase is substantially greater than the genome-wide LOH increases observed in the *pol32* mutant compared to wild type (Table 1).

We then examined the breakpoints of LOH in *pol32* and wild-type strains exhibiting the 5-FOA^R^ His^+^ phenotype. This analysis for the wild-type strain was done using SNP-specific microarrays that are capable of detecting LOH with a resolution only slightly less than by genomic sequencing (21). Surprisingly, in 14 5-FOA^R^ His^+^ colonies from the *pol32* strain, only 4 were single-transition T-LOH events. Two-thirds had multiple transitions (Fig. S3). For most events, the recombination-initiating lesions were more than 100 kb from the 65-kb hotspot. In contrast, in the control wild-type strain, 9 out of 11 were simple LOH events with the recombination-initiating lesion near or within the hotspot (Fig. S4), a statistically significant (*P* = 0.015, Fisher’s exact test) difference from the *pol32* data. This result suggests that repair of DSBs at the hotspot in *pol32* strains often reflects multiple transitions between different templates or that the hotspot region is often associated with multiple concerted recombinogenic lesions.

As in previous experiments with other types of genome-destabilizing mutants (20), we determined whether the breakpoints of recombination events were non-randomly associated with chromosomal elements (e.g., replication origins and repetitive sequences). This analysis is summarized in Data set S1-3. Replication termination sequences and weakly transcribed genes were under-represented at LOH breakpoints, whereas snRNA genes and highly transcribed genes were over-represented. These associations were not well conserved with those found in wild-type strains (23) or in strains with low levels of replicative DNA polymerases (18–20).

### Elevated levels of large deletions/duplications, translocations, telomere length alterations, and aneuploidy in *pol32* diploids

**Fig 3:**
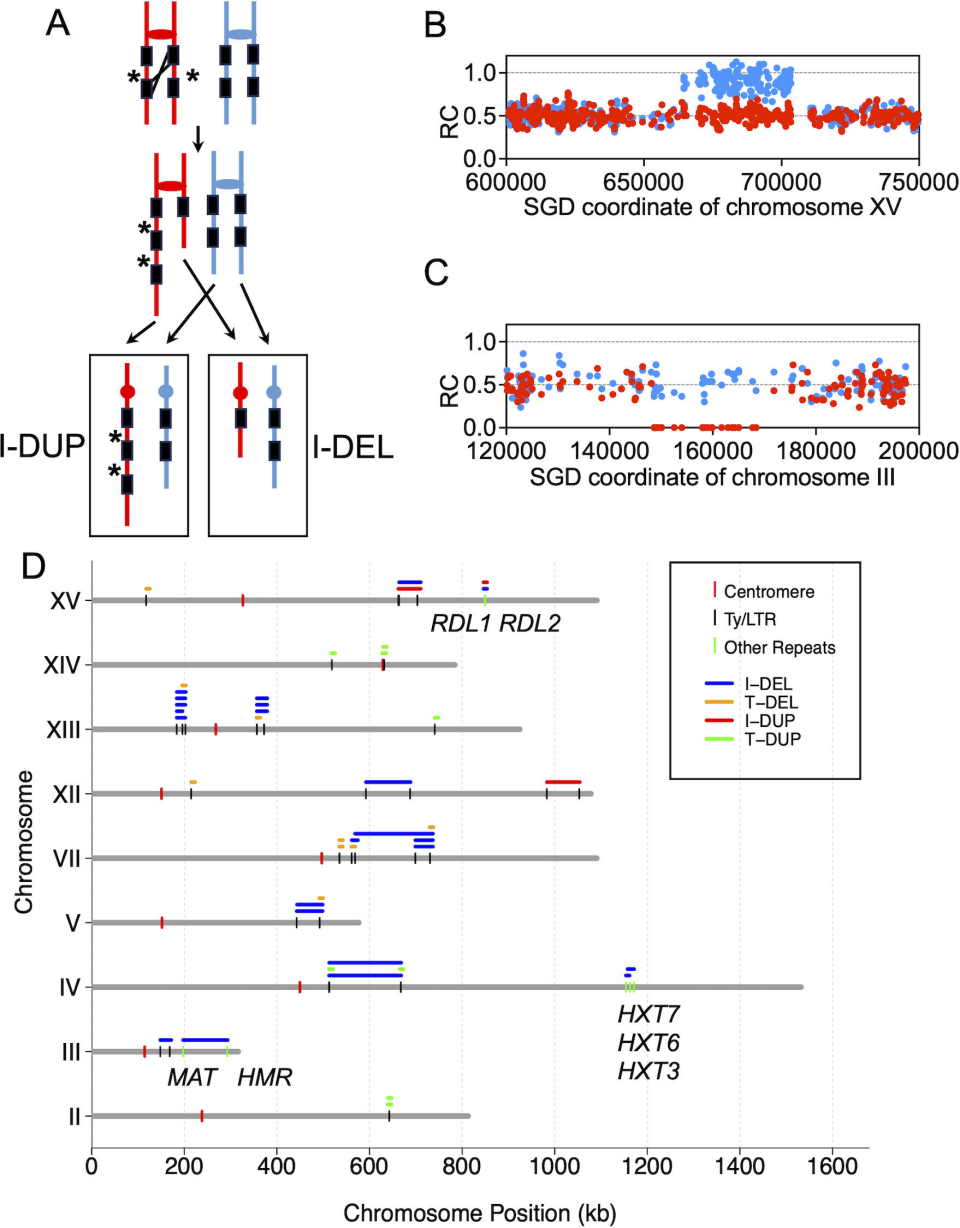
Large insertions/deletions resulting from recombination between non-allelic repeats. (**A**) Unequal recombination between direct repeats on sister chromatids results in duplication or deletion of intervening sequences. The region separating the two repeats on the red homolog is shown with asterisks. A reciprocal crossover can produce one daughter cell with a duplication of the intervening region and one with a deletion. Deletions could also be generated by single-strand annealing caused by a DSB between the two repeats (not shown). (**B**) Interstitial duplication (I-DUP). These events are characterized by a segment in which one set of SNPs has an RC of 1 and the other set has an RC of 0.5. The duplicated region is flanked by heterozygous sequences. (**C**) Interstitial deletion (I-DEL). I-DEL events have a contiguous set of SNPs derived from one homolog with an RC of 0 and SNPs derived from the other homolog of 0.5. (**D**) Distribution of large insertions/deletions on yeast chromosomes. The deletions and duplications are shown as horizontal lines above each chromosome using the color code indicated on the figure. The short black vertical lines represent Ty or delta elements; most are Ty elements (Data set S1-4). Short green vertical lines represent other types of repeats.

Large interstitial deletions and duplications (I-DEL and I-DUP, respectively) can be produced by HR between repeated genes located at different positions on sister chromatids (Fig. 3A) (20, 30). Recombinations between genes repeated on non-homologous chromosomes can result in translocations that have paired terminal deletions (T-DEL) and duplications (T-DUP) within one isolate. Among the 46 WYpol32-derived isolates, we observed 43 large (>5 kb) deletions/duplications (Table 1; Data set S1-4; Fig. 3A). Examples of I-DUP and I-DEL events are shown in Fig. 3B and C. The rates of the interstitial and terminal deletions/duplications are 16- and 52-fold higher than observed in wild-type cells (23). Almost all of these rearrangement classes had repetitive sequences of the same type at the rearrangement breakpoints, including the transposable elements Ty1/2, *HXT7/6/3*, and *MAT* loci (Data set S1-4; Fig. 3D). This finding indicates that those chromosomal rearrangements reflect ectopic HR, recombination between non-allelic repeats.

**Fig 4:**
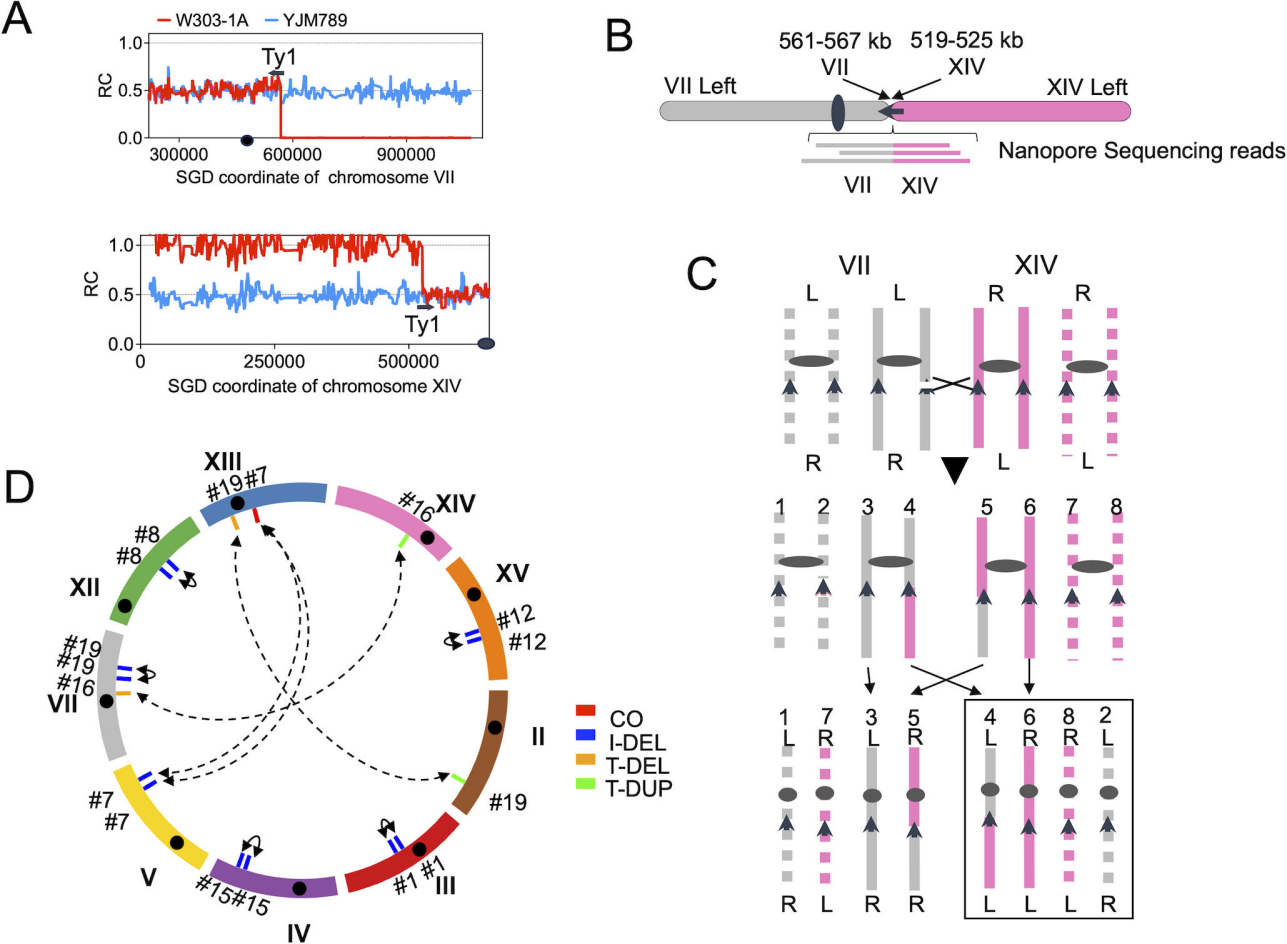
Analysis of deletions and duplications based on DNA sequencing in WYpol32 isolates. (**A**) Sequence coverage of SNPs from chromosomes VII and XIV in WYpol32-8-16. The breakpoints are near coordinate 561–567 kb on the right arm of VII and near coordinate 519–525 kb on the left arm of XIV. Chromosome VII has a Crick-oriented Ty1 element between coordinates 561–567 kb, and a Watson-oriented Ty1 element is on the left arm of XIV at coordinates 519–525 kb. The black circles show the approximate position of the centromeres. (**B**) Nanopore DNA sequencing analysis. By long-range DNA sequencing, we detected “reads” that spanned the breakpoint between VII and XIV. (**C**) Mechanism for generating the translocation. Chromosomes VII and XIV are in gray and pink colors, respectively. Non-dotted and dotted lines indicate that the chromosomes were derived from W303 and YJM789, respectively. Arrows show Ty1 elements. L and R designate the left and right arms of the chromosome. The translocation results from a crossover between Ty1 elements on homologs derived from W303. Following segregation of the chromosomes, one daughter cell (outlined) has a terminal duplication of W303-derived sequences centromere-distal to the Ty element on the left arm of XIV, and a terminal deletion of W303-derived sequences centromere-distal to the Ty element on the right arm of VII. (**D**) Chromosome rearrangements confirmed by long-read DNA sequencing. Blue, orange, and green show I-DEL, T-DEL, and T-DUP events, respectively. Red indicates a crossover. The dashed double-headed arrows show translocations, and the solid arrows show I-DEL events. The “#” designation on the outside of the circle corresponds to the number of the WYpol32 isolates shown in Data set S1. The crossover event of WYpol32-8-7 is described in detail in Fig. S5. All alterations have repeated genes at the junctions of the deletions or duplications, as indicated by the short vertical lines on each chromosome. The black circles represent centromeres, and each chromosome is oriented from the left arm to the right arm in a clockwise direction.

The T-DEL/DUP events typically occur in pairs in the same isolate. For instance, in isolate WYpol32-8-16, we observed a T-DEL beginning at *Saccharomyces* Genome Database (SGD) coordinate 561 kb and extending to the right end of chromosome VII; this deletion was accompanied by a T-DUP from the left end to 525 kb of chromosome XIV (Fig. 4A). Nanopore sequencing confirmed this translocation (Fig. 4B). Figure 4C shows how ectopic recombination between Ty elements on non-homologous chromosomes could produce the observed deletions and duplications. Figure 4D shows the recombinations of 10 other duplications and deletions that were confirmed by Nanopore sequencing. Other than the “paired” terminal DEL/DUP, Figure S5 revealed that the apparent I-DEL on chromosome V actually resulted from two Ty-mediated ectopic recombination events producing balanced translocations between chromosomes V and XIII.

The rDNA locus in *S. cerevisiae* consists of many tandemly repeated 9 kb units located on chromosome XII (31). In our genetic background, wild-type diploid isolates grown on rich medium, the median number of rDNA repeats was 186 (95% confidence limit [CL] = 182–194) (23). The WYpol32-derived isolates exhibit a median rDNA copy number of 134 (95% CL = 119–155; Fig. S6A), a significant reduction compared to wild type (*P* < 0.0001, Mann-Whitney test). In agreement with prior studies of reduced DNA polymerase activity, these results suggest that rDNA copy number loss is driven by DNA replication stress (18–20). Similar to the tandemly repeated rDNA loci, deletion of *POL32* also significantly reduced the copy number of the *CUP1* array (32) compared to the wild-type strain (Fig. S6B; *P* < 0.001, Mann-Whitney test) (23).

In *S. cerevisiae*, telomeres are composed of short imperfect repeats of the form 5′-TG_1-3_-3′ (33, 34). By nanopore sequencing of 15 wild-type (23) and 8 *pol32* isolates, we found that telomeres at each chromosome end were longer in *pol32* strains than in wild-type cells. The median telomere length was 362 bp in *pol32* strains (95% CL = 341–379, Fig. S6C) and 311 bp in wild-type cells (95% CL = 299–332). This increase in length was significant (*P* < 0.0001, Mann-Whitney test), confirming the moderate telomere elongation phenotype previously described for *pol32* strains (35).

Aneuploidy is common in WYpol32-derived isolates, occurring at a rate about 50-fold greater than in wild-type strains (Table 1). The aneuploid events (Fig. S6D) include 12 monosomes (2*N* − 1), 13 trisomes (2*N* + 1), and 1 tetrasome (2*N* + 2) (Data set S1-5). In addition, we observed six examples of uniparental disomy (loss of one homolog and duplication of the other; UPD).

### Effects of the *pol32* mutation on the rate and spectrum of mutations

Previous studies indicated that *pol32* strains had a slightly lower rate of spontaneous mutations (twofold [6, 36]), or slightly higher or lower rates dependent on the genetic background (12). Most of these rates were estimated using the *CAN1* gene rather than by whole-genome analysis.

By whole-genome sequencing, we found 89 mutations (Data set S1-6) in WYpol32 isolates, a rate about twofold greater than the wild type (Table 1). Using the *CAN1* assay, we also found that the *pol32* mutant exhibited a 1.7-fold higher mutation rate than the wild type. These results confirm that the *pol32* mutation has a slightly elevated rate of SNVs in our genetic background.

The rate of small (<20 bp) in/dels was about fivefold higher than the wild type (Table 1). Most of the in/dels (12 out of 14) occurred within mononucleotide tracts or microsatellite regions, while the remaining two in/dels were flanked by short direct repeats (Data set S1-6). These small in/dels were likely a result of DNA polymerase slippage, suggesting that primer-template strand dissociation is more pronounced in the *pol32* mutant strains than in the wild-type strains.

**Fig 5:**
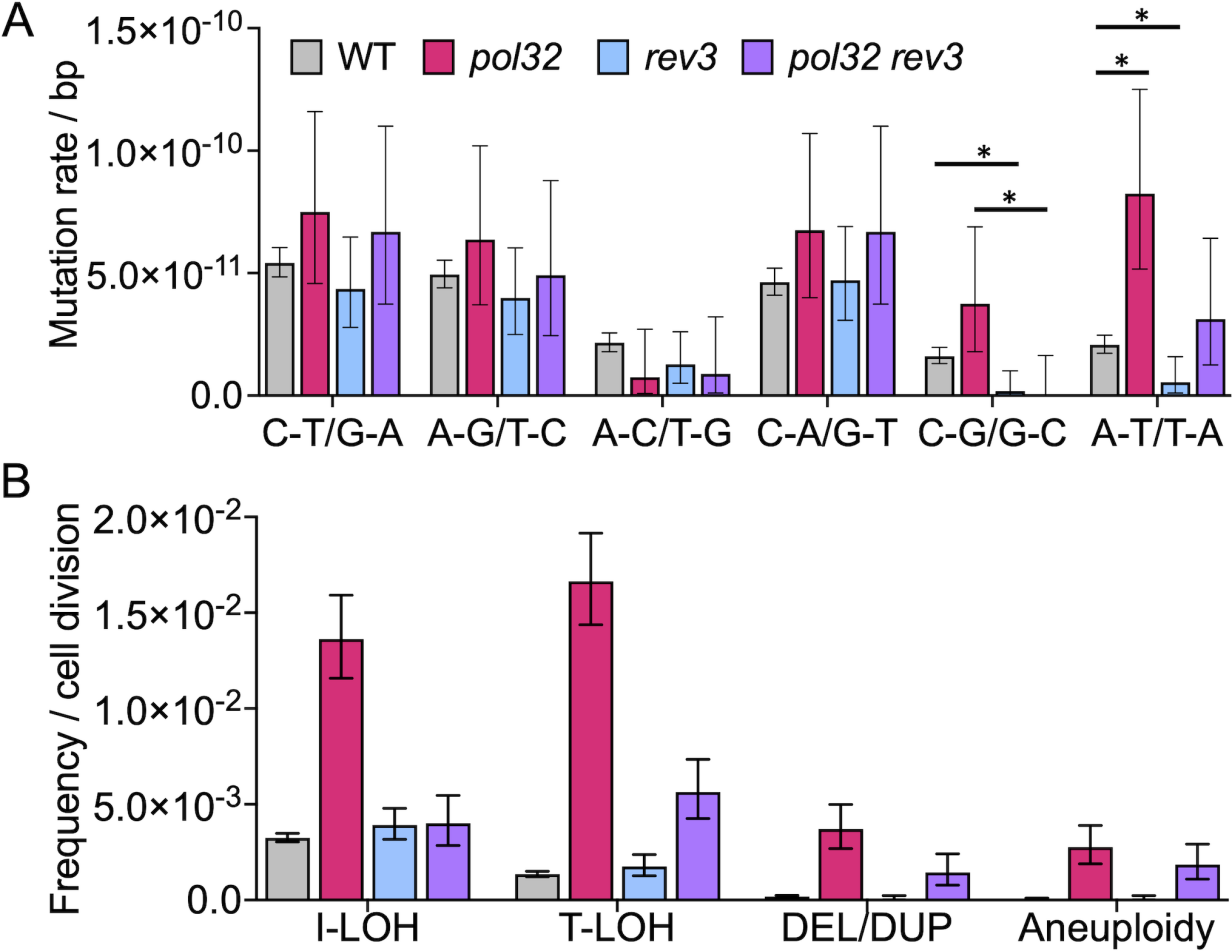
Mutation spectra and frequencies of genomic alterations in *pol32*, *rev3*, and *pol32 rev3* strains. (**A**) Mutation rate of base substitutions detected in wild-type (WT), *pol32*, *rev3*, and *pol32 rev3* strains. The asterisk denotes a statistically significant (*P* < 0.05) difference from the wild type. (**B**) Frequencies of interstitial loss of heterozygosity (I-LOH), terminal loss of heterozygosity (T-LOH), deletions/duplications (DEL/DUP), and aneuploidy events per cell division in WT, *rev3*, *pol32*, and *pol32 rev3* strains. Error bars represent the 95% confidence limit (CL).

The *POL32* deletion also altered the spectrum of single-base changes. In wild-type cells, A to T/T to A transversions accounted for approximately 10% of all base substitutions, occurring at a rate of 2.1 × 10^−11^ per base pair per cell division (23). In *pol32* strains, this mutation class occurred at a fourfold higher rate and represented about 25% of all substitutions (22 out of 89, Data set S1-6; Fig. 5A; *P* < 0.001 by Fisher’s exact test). An elevation of this type of mutation has been observed previously in yeast strains with mutations affecting DNA polymerases or polymerase co-factors (20, 37, 38).

### Elevated rates of reciprocal crossovers in *pol32* strains: evidence that these events are initiated by DSBs formed during the S-period

Although the analysis described above showed that *pol32* strains had an elevated rate of T-LOH, it was not clear whether these events reflected reciprocal crossovers or BIR events. We previously described a colony-color screen that allowed unambiguous detection of reciprocal crossovers on the right arm of chromosome IV (21, 22). Using this method (described in Supplemental information; Data set S2; Fig. S7), we found that the *pol32* mutation resulted in a crossover frequency of 5 × 10^−4^/division, about 16-fold higher than observed in the wild-type cells. Further, by examining the patterns of gene conversion events associated with the crossovers, we found that about 36% and 64% of the crossovers are initiated in G1 and S, respectively. In contrast, for the wild-type strain, most (70%) spontaneous crossovers are initiated in G1 (22).

### Role of Rev3 in *pol32*-induced genetic alterations

Since Pol32 functions as an accessory subunit for Pols δ and ζ, the widespread chromosomal instability observed in the *pol32* mutant may arise from defects in one or more of these DNA polymerases. To examine the role of Pol ζ in genome stability, we analyzed two diploids, WY75 (*rev3*) and QL60 (*pol32 rev3*). Following 60 cycles of sub-cloning, we sequenced 16 independent isolates of WY75. The rate of SNVs was decreased in the *rev3* strain by about 40% (Data set S3; Table S3), consistent with previous observations (39). The deletion of *REV3* reduced mutation rates for most substitution classes relative to wild type (23). In particular, the A to T/T to A and C to G/G to C substitutions in *rev3* strains were less frequent than in the wild-type strain (*P* < 0.05, Poisson rate test; Fig. 5A) (23).

The rates of various classes of genome instability in the *rev3* mutant were not significantly different from wild type (Fig. 5B) (23). Also, the proportions of I-LOH (69%) and T-LOH (31%) in the *rev3* mutant were similar to wild type but differed significantly from *pol32* isolates (*P* < 0.0001, Fisher’s exact test; Table S4).

Although the *rev3* mutation had little effect on most types of genetic alterations compared to the wild-type strain, the *pol32 rev3* strain (QL60, 13 isolates that underwent 30 rounds of sub-culturing) had significantly reduced the rates of most genomic changes relative to the *pol32* strain (Fig. 5B; Table S3). Significant reductions were observed for I-LOH, T-LOH, deletions, and SNVs (Fig. 5B). In addition, although the rates of I-LOH and SNVs in the *pol32 rev3* strain were reduced to levels similar to the wild-type strain, other classes were still elevated compared to the wild-type strain (Table S3). These results demonstrate that the genomic instability in *pol32* strains is partly dependent on Rev3.

## DISCUSSION

This study systematically investigates the genome-wide effects of deleting *POL32* in diploid strains of *S. cerevisiae*, revealing unique roles of this DNA polymerase subunit in maintaining genome integrity. Below, we compare the genetic instability induced by loss of Pol32 with the instability observed in the wild-type strain and in strains under replication stress. We also discuss the role of the error-prone DNA polymerase ζ in elevating the genomic instability of *pol32* strains.

### Patterns of genomic rearrangements in *pol32* diploids: comparisons with the wild-type strain and with strains with low DNA polymerase levels

Since Pol32 is a subunit of DNA polymerases δ, we expected the phenotypes of the *pol32* deletion to mimic the genomic instability observed in strains with low levels of Pol δ. In general, this expectation was confirmed, although the quantitative effects of the *pol32* mutation were smaller. It is likely that the relevant level of genomic alteration is a consequence of increased DSBs, reflecting increased fragility of the replication forks. This conclusion is supported by the increase in S/G2-initiated recombination events in *pol32* strains, similar to that observed in strains with low levels of the replicative DNA polymerases (19).

In wild-type strains, I-LOH events are at least twofold more frequent than T-LOH events (23, 40). In contrast, in the *pol32* strain and in the strains with low levels of replicative DNA polymerases, T-LOH events exceed I-LOH events (18–20). Since both types of events are likely initiated by DSBs, this result argues that the replication stress not only changes the level of recombinogenic lesions, but also affects the repair of lesions. Most spontaneous events are initiated by DSBs formed in G1/G0 of the cell cycle, whereas those in strains with low replicative DNA polymerases are generated in S (30). This difference may affect the processing of broken DNA ends and the subsequent use of these ends in the crossover pathway (producing T-LOH) and in the I-LOH pathway (producing gene conversion events without an associated crossover). Although we currently cannot offer a definitive explanation of the interesting difference between *pol32* and wild type, the similarity between *pol32* and the strains with low polymerase argues that the effect may be related directly or indirectly to replication stress.

In general, the *pol32* strain had elevated rates of most categories of genome alterations, but had smaller effects than those associated with low levels of DNA polymerases α, δ, or ε (19). One exception to the similarity of the genome-destabilizing effects of the low-polymerase strains and the effect of *pol32* is the rate of SNVs. All low-polymerase strains exhibit at least a 10-fold elevation in SNV rates. This mutator phenotype is thought to arise from stalled replication forks or increased single-stranded DNA that recruit error-prone polymerases such as Pol ζ to drive mutagenesis. Strains with reduced levels of replicative polymerases display a relative increase in G to C/C to G substitutions (19); this preference has been shown to be dependent on Pol ζ (41). In contrast, *pol32* strains show less than a twofold elevation in mutation rates. Since Pol32 is a subunit of Pol ζ, the reduced activity of this error-prone polymerase may counterbalance the mutation-promoting effects of replication defects in *pol32* mutants, resulting in a milder overall mutator phenotype. The only significant shift in the substitution spectrum in *pol32* strains is an increase in A to T/T to A substitutions compared to wild-type. This bias is not prominent in low-polymerase strains but parallels observations in *met18* mutants (38). Further supporting Pol ζ involvement, the *rev3* deletion reduces both G to C/C to G and A to T/T to A ratios relative to wild type (Fig. 5A), confirming that both substitution classes are linked to Pol ζ function. Collectively, these analyses suggest that Pol32 and Fe-S clusters are required for Pol ζ-mediated G to C/C to G substitutions but are dispensable for its induction of A to T/T to A substitutions, revealing distinct mechanistic requirements for Pol ζ-dependent mutation signatures (19, 38).

As with the low-polymerase strains, the *pol32* strain has a substantially elevated rate of aneuploidy compared to the wild-type strain. This increase can be caused by decreased repair of DSBs (leading to monosomy) as observed in *rad52* strains (42). Alternatively, slower replication in the *pol32* strains may result in partially replicated chromosomes. If these chromosomes are segregated into daughter cells, both monosomes and trisomes could be generated.

For the low-polymerase strains (18–20), the ratios of monosomes to trisomes were: 12 (low Pol α), 4.6 (low Pol δ), and 2.4 (low Pol ε), suggesting that aneuploidy was a consequence of failure to repair DSBs or a failure to replicate one of the homologs. In contrast, the ratio of monosomy to trisomy in the *pol32* strain was close to 1, indicating that non-disjunction or the segregation of incompletely replicated chromosomes was responsible for the aneuploidy.

### A recombination hotspot and a cold chromosome in *pol32* strains

LOH events were distributed differently in the *pol32* strain from other strains we have examined. We observed a region near the end of chromosome VII in *pol32* strains that had a significantly higher frequency of LOH breakpoints than other regions of the genome (Fig. 2; Fig. S2). Recombination events selected in this region had multiple switches between LOH regions derived from different homologs (Fig. S3); this pattern was not observed in wild-type strains (Fig. S4). Two possibilities for generating multiple LOH switches between different homologs are shown in Fig. S8. First, it is possible that very long symmetric heteroduplexes are formed between unreplicated chromosomes. Patchy repair of such heteroduplexes could produce multiple LOH switches (Fig. S8A). Alternatively, delayed replication of chromosome VII in *pol32* strains could produce lagging chromosomes during segregation that acquire multiple DSBs on both homologs; such a phenomenon resembles chromothripsis of mammalian chromosomes (42). Subsequent DNA repair events could produce the mosaic pattern of LOH (Fig. S8B).

Chromosome IX had significantly fewer events expected from a random distribution (Fig. 2C). In addition, the pattern of reciprocal crossovers on chromosome IV is different in wild-type and *pol32* strains, with the *pol32* strain having more events within 200 kb of the centromere (Fig. S9).

As described in the Results section, we found that the breakpoints of LOH events in the *pol32* strains were significantly associated with highly transcribed genes and snRNA genes and were significantly depleted for replication-termination sequences and weakly transcribed genes (Data set S1-3). Therefore, we determine whether the 65 kb “hot” region on VII or the “cold” chromosome IX was enriched or depleted for these chromosome elements. No significant associations were observed. In summary, our study suggests that DSB formation in *pol32* strains may be sensitive to some element (as yet undefined) of chromosome structure, perhaps related to the timing of replication in *pol32* strains. Alternatively, the *pol32* mutation may result in a regional or a chromosome-specific preference for a DSB to be repaired using the homolog or the sister chromatid as a template.

### The role of Rev3/Pol ζ in the genomic rearrangements formed in *pol32* cells

To examine the role of Rev3 in influencing the rate of genomic changes observed in *pol32* strains, we examined the rates of changes in *rev3* (WY75) and *pol32 rev3* (QL60) homozygous diploids (Table S3). The single *rev3* mutation had no significant effect on the rates of LOH, large deletion/duplication, or aneuploidy (Fig. 5). SNV rates were slightly, but significantly, reduced in the *rev3* strain relative to the wild-type strain, as expected from previous studies (39).

Although the *rev3* mutation has little effect on genome stability, the *rev3 pol32* double mutant strain showed significantly lower (two- to three-fold) rates than the *pol32* single mutant (Fig. 5B; Table S3). Despite these reductions, the rates of most genome rearrangements in the *rev3 pol32* strains were still above the rates observed in wild type (Table S3). These results taken together argue that the genome instability observed in *pol32* strains is partly dependent and partly independent of DNA Pol ζ. Although this observation has a number of interpretations, one possible explanation is that the primary genome-destabilizing effect of the *pol32* mutation is a reduction in the activity of the replicative DNA polymerase δ. If there is a competition between Pol δ and the error-prone Pol ζ at the replication fork, loss of Pol ζ may allow the partially defective replicative DNA δ to function more efficiently, reducing genome instability. Alternatively, in strains undergoing replication stress because of the *pol32* mutation, Pol ζ may introduce elevated levels of recombinogenic DNA lesions.

### Summary

Pol32 serves as a key guardian of genome stability in yeast, affecting DNA replication, DNA repair, and recombination pathways. Its depletion elevates multiple types of genomic alterations, producing a pattern of instability that is different from that observed in strains with reduced levels of replicative DNA polymerases. Our results highlight the importance of polymerase accessory subunits in genome maintenance and inform our understanding of genome instability linked to Pol32/POLD3 dysfunction in human disease.

## MATERIALS AND METHODS

### Strains and medium

The genotypes of yeast strains used in our study and details of the strain constructions are shown in Tables S1 and S2. We used the LiAC/PEG/ssDNA method for yeast transformation (43). Standard recipes for media such as YPD (yeast extract peptone dextrose) were used (44).

### Whole-genome sequencing and SNP microarray analyses

We used both short-read Illumina and long-read Nanopore sequencing in our studies. The sequencing and analysis were performed as described in previous studies (23, 45); detailed procedures are presented in the supplemental materials.

For microarray analysis, genomic DNA extracted from sectors and control colonies was labeled with Cy5-dUTP and Cy3-dUTP, respectively. The two labeled DNA samples were competitively hybridized onto SNP microarrays and the hybridization ratios were analyzed as described previously (21).

## Data Availability

The raw sequencing data were deposited in SRA database (https://www.ncbi.nlm.nih.gov/bioproject/?term=PRJNA1233794). The raw SNP microarray data were deposited in the Gene Expression Omnibus (GEO) database (https://www.ncbi.nlm.nih.gov/geo/query/acc.cgi?acc=GSE291566) and ArrayExpress (https://www.ebi.ac.uk/biostudies/ArrayExpress/studies/E-MTAB-16051?query=E-MTAB-16051).

## SUPPLEMENTAL MATERIAL

Data set S1 mbio.00531-26-s0001.xlsx Genomic alteration events in WYpol32 isolates cultured at 30°C.

Data set S2 mbio.00531-26-s0002.xlsx Mitotic recombination events on chrosomome IV in WYpol32 red/white sectors.

Data set S3 mbio.00531-26-s0003.xlsx Genomic alteration events in WY75 (*rev3*) and QL60 (*pol32 rev3*) isolates sub-cultured at 30°C.

Supplemental Information mbio.00531-26-s0004.docx Supplemental text, figures, and tables.

ASM does not own the copyrights to Supplemental Material that may be linked to, or accessed through, an article. The authors have granted ASM a non-exclusive, world-wide license to publish the Supplemental Material files. Please contact the corresponding author directly for reuse.
