# Supplementary material for: Loss of Pol32, a subunit of DNA polymerases δ and ζ, leads to different patterns of genome stability than direct impairment of these individual polymerases
Source: mBio. 2026 Apr 17;17(5):e00531-26. doi: 10.1128/mbio.00531-26 (PMC13170350; doi:10.1128/mbio.00531-26)
Supplement: Supplemental Information — Supplemental text, figures, and tables. [file mbio.00531-26-s0004.docx]

**Supplemental information**

**Loss of Pol32, a subunit of DNA polymerases δ** **and** **ζ**, **leads to different patterns of genome stability than direct impairment of these individual polymerases**

Lei Qi^1,2^, Ke-Jing Li^1^, Xing-Xing Tang^1,2^, Li-Yan Tian^1^, Ye-Ke Wang^3^, Min He^1^, Ke Zhang^3*^, Thomas D. Petes^2*^, Dao-Qiong Zheng^1*^

^1^ State Key Laboratory (SKL) of Biobased Transportation Fuel Technology, Ocean College, Zhejiang University, Zhoushan, 316021, China

^2^ Department of Molecular Genetics and Microbiology, Duke University, Durham, NC, 27710, USA

^3^ College of Life Science, Zhejiang University, Hangzhou, 310058, China

*Corresponding author: zhengdaoqiong@zju.edu.cn, [tom.petes@duke.edu](mailto:tom.petes@duke.edu), or zhangke726@zju.edu.cn

**Supplemental Text.** Strain constructions, method details, supplemental figure legends and supplemental references.

**Supplemental Tables:**

Table S1. Strain constructions and strain list.

Table S2. Primers used in this study.

Table S3. Numbers and rates of genome alterations in *pol32, rev3,* and *pol32 rev3* diploids.

Table S4. Ratio of LOH events that are I-LOH and T-LOH for strains of different genotypes.

**Supplemental Figures:**

Figure S1. Detection of DSBs in wild-type and *pol32* strains using a fluorescently-tagged Rad52 protein.

Figure S2. Analysis of chromosome VII hotspot.

Figure S3. Patterns of LOH at the chromosome VII hotspot in *pol32* isolates (DNA sequence analysis).

Figure S4. Patterns of LOH at the chromosome VII hotspot in wild-type isolates (microarray analysis).

Figure S5. I-DEL event in isolate WYpol32-8-7 resulting from non-allelic recombination events that produce two translocations.

Figure S6. Effect of the *pol32* mutation on the number of copies of ribosomal DNA (rDNA) and *CUP1* sequences, telomere length, and chromosome disjunction.

Figure S7. Patterns of mitotic recombination detected by analyzing samples derived from sectored colonies.

Figure S8. Mechanisms that could produce multiple switches of LOH regions such as those observed in the hotspot for LOH events on the right arm of chromosome VII.

Figure S9. Location of breakpoints for reciprocal crossovers on chromosome IV in wild-type and *pol32* strains.

**Strain Constructions**

Most of the diploids used in our study were derived from two haploids (W303 and YJM789) used in previous studies of mitotic recombination. The diploids used in this analysis were heterozygous for about 50,000 SNPs, allowing high-resolution mapping of genomic rearrangements throughout the genome. The details of the constructions are given in Table S1 and the primers used for these constructions are listed in Table S2

**Detection of Rad52 foci**

To detect Rad52 foci in the wild-type and *pol32* mutant strains (Fig. S1), we imaged yeast strains expressing Rad52 fused with YFP (described in Table S1) under a high-resolution confocal microscope (OLYMPUS FV3000 Japan). The yeast cells were incubated in 5 ml liquid YPD to log phase (OD_600_ of 0.1). Rad52-YFP foci in Z-image stacks were acquired and counted. Independent experiments were performed three times to get the mean values. X8068-6C and X8068-6Cpol32 were used as the wild-type and *pol32* mutant strains, respectively, for Rad52 foci analysis.

**Illumina DNA sequencing analysis**

For the short-read analysis, genomic DNA of yeast cells was extracted using the Omega yeast DNA kit (Life Science Products). Whole-genome sequencing and subsequent analysis were performed as described in our previous studies (1). Briefly, paired reads (2×150 bp) were sequenced to an average sequencing depth of 120× using the Illumina NextSeq 500 platform. The reads were mapped on the S288C reference genome using BWA (2) to obtain .sam files (2), which were then converted to sorted .bam files using Samtools (3). The .bam files were processed by Samtools (3) and VarScan 2 (4) to determine sequencing coverage and detect mutations.

**Nanopore sequencing and analysis**

Nanopore sequencing of the yeast genome was done as described in (5). Genomic DNA was extracted as described by (6) with the exception that zymolyase was used rather than lyticase. The genomic DNA samples were barcoded with the EXP-NBD104 kit (Oxford Nanopore Technology), and a pooled sequencing library was prepared using the SQK-LSK109 kit (Oxford Nanopore Technology). After purification, 700 nanograms of the DNA libraries were loaded onto a MinION flow cell (FLO-MIN106D R9.4.1, Oxford Nanopore Technology) and sequenced on a MinION device. Real-time sequencing and base-calling were managed by MinKNOW (v21.02.1) with the built-in Guppy (v4.3.2) basecaller. Long reads were aligned to the S288C genome using NGMLR software (7). The .sam files were converted into sorted .bam files using Samtools (3). Structural variations were subsequently identified with Sniffles (7), generating .vcf files.

**Analysis of genomic alterations by short-read sequencing of isolates from mutation-accumulation experiments.**

Most of our experiments utilized short-read Illumina sequencing to analyze the genetic alterations induced in *pol32* strains during mutation accumulation experiments. In these experiments, the average coverage of genomic sequences was >100. The most abundant classes of alterations were interstitial LOH (I-LOH) and terminal LOH (T-LOH). The definition of I-LOH was an event in which one or more of the heterozygous SNPs was duplicated for one allele and deleted for the other allele. The criteria used to determine deletions or duplications was based on the ratio of coverage (RC), the number of “reads” for each heterozygous SNP divided by the average number of “reads” for all SNPs in the genome. A deletion required the RC to be less than 0.05, whereas the duplication was defined as an RC > 0.7.

Most I-LOH events included several SNPs and, in most of these multi-SNP events, alleles derived from the same homolog were duplicated or deleted (Classes a1 and a2 in Dataset S1-2). However, we also observed complex I-LOH events (labeled c1-c26 in Dataset S1-2). In such tracts, the I-LOH region was interrupted by one or more regions of heterozygous SNPs or regions of LOH derived from two different donor chromosomes were contiguous. For example, in Class c4, two regions of I-LOH were separated by one or more SNPs that were heterozygous. If the two regions of I-LOH were separated by less than 20 kb, they were counted as a single event. This criterion (which have used previously) was based on previous observations that single mitotic gene conversion events could extend to 20 kb and these long conversion tracts were sometimes interrupted by heterozygous regions, likely the result of patchy mismatch repair within the heteroduplex formed as an intermediate in gene conversion (8). In addition to I-LOH events in which regions of LOH are interspersed with regions of heterozygosity, we found I-LOH events in which there are regions of homozygosity from two different donors (for example, Class c3 in Dataset 1-2). Such events were also classified as single I-LOH regions if the transitions were within 20 kb. If I-LOH regions were separated by more than 20 kb, they were considered two separate events.

T-LOH regions were usually events with a single breakpoint separating the heterozygous portion of the chromosome from an LOH region that extended to the end of the chromosome (Classes b1-b4 in Dataset S1-2). As observed with the I-LOH events, however, we also found complex T-LOH events in which the LOH region was interrupted by one or more transitions (Classes d1-d42). As with the I-LOH events, if these transitions were separated by less than 20 kb, they were considered part of a single events; if separated by more than 20 kb, they were considered independent events. Complex events may reflect patchy repair within a long heteroduplex, break-induced repair (BIR) events that involve multiple switches between different templates and/or clustered DNA breaks (8).

In addition to I-LOH and T-LOH events, we detected numerous other chromosome alterations. Large interstitial duplications (I-DUP) resulted in regions with an RC of 0.7 for one or more SNPs coupled with an RC value of about 0.5 for the SNPs derived from the other homolog (Fig. 3B). I-DEL (large interstitial deletions) are events in which one or more SNPs had an RC less than 0.05 and the allelic SNPs had an RC of near 0.5 (Fig. 3C). Terminal large duplications and deletions (T-DUP and T-DEL) are classified in a similar way except the duplicated and deleted regions extend to the end of the chromosome. Isolates that contain both terminal deletions and terminal duplications often represented translocations (Fig. 4).

We also detected aneuploidy. In monosomic strains, the RC for all SNPs from one homolog were reduced to less the 0.05, and the RC for the other homolog remained at about 0.5. In trisomic strains, all SNPs from one homolog were elevated to an RC > 0.7 and the RC from the other homolog remained at about 0.5. Lastly, we detected uniparental disomy (UPD) for some chromosomes. UPD is characterized by all SNPs from one homolog at an RC value of less than 0.05, and all SNPs from the other chromosome at an RC value > 0.7.

In addition to the chromosome alteration described above, short-term sequencing readily detected single-base mutations and short (< 50 bp) additions and deletions. Since these mutations occurred in diploids, approximately half of the reads had the mutant alteration and half the original sequence. Pre-existing mutations were excluded from the analysis and any mutation that appeared in more than three isolates was excluded as likely representing a variant that occurred before the mutation-accumulation experiment was initiated.

**Analysis of genomic alterations by long-read sequencing of isolates from mutation-accumulation experiments.**

We performed long-read (Nanopore) sequencing on eight isolates of *pol32*. In addition to confirming the results found with short-read sequencing, this analysis demonstrated the translocation between chromosomes VII and XIV (Fig. 4B) and a number of other chromosome rearrangements involving ectopic recombination between non-allelic Ty1 elements (Fig. 4D). Long-reads are useful for this purpose because they allow detection of non-allelic genomic sequences that span the 6 kb Ty elements. This analysis cannot be performed with short-read Illumina sequencing in which the individual reads are only several hundred bp in length.

**Analysis of mitotic recombination events on chromosome IV detected in sectored colonies derived from *pol32* diploids**

Using a colony-color screening system, we characterized the patterns of reciprocal crossovers on chromosome IV in *pol32* diploid (Fig. S7). WYpol32 colonies were diluted and spread onto YPD plates, then incubated at 30°C for 3 days. Red/white sectored colonies were analyzed using custom chromosome IV-specific SNP microarrays (8). This system allows us to infer whether the recombinogenic DNA lesion was generated before or after DNA replication (9, 10). Many crossovers are associated with an adjacent region of gene conversion (11). If the initiating lesion was formed during or after DNA replication on one chromatid in the diploid, considering both sectors, we expect to see a 3:1 conversion (Fig. S7A). If the initiating lesion occurred on an unreplicated homolog that was then replicated to produce two broken chromatids (Fig. S7B), we expect that the two sectors will have a 4:0 conversion event adjacent to the crossover. If the repair events of the two broken chromatids involve different extents of heteroduplex formation (Fig. S7C), 4:0/3:1 hybrid conversions are expected (10).

The frequency of sectored colonies was 5 × 10^-4^ (20/39835) in the *pol32* mutant, about 16-fold more than the frequency of sectoring in an isogenic wild-type strain (10). We analyzed both sectors of 40 red/white sectored colonies using SNP microarrays (8, 10); the resolution of this method is similar to that obtained by genomic sequencing. An example of this analysis is shown in Fig. S7C and S7D.

The patterns and breakpoints of the recombination events in the sectored colonies are shown in Dataset S2. Using the criterion described above to diagnose whether the event was initiated in G1 or S/G2, 9 were initiated in G1, 16 were initiated in S/G2, and 15 were ambiguous (no conversion event associated with the crossover). Considering only those sectors in which crossovers were associated with gene conversions, 36% were the result of G1-initiated events and 64% were initiated in G2/S. In our previous studies, we showed that spontaneous events were usually initiated in G1 (70%; (10)), whereas strains with low DNA polymerases α, δ, or ε were found to preferentially initiate recombination in S/G2 (> 80%) (reviewed by (12)). Thus, the preference for recombinogenic lesions initiating in S/G2 observed in the *pol32* diploids suggests that the elevated rate of mitotic recombination reflects a partial defect in DNA replication. In Fig. S9, we show the locations of breakpoints on chromosome IV were enriched between coordinates 1 Mb and 1.2 Mb. A similar distribution was previously observed in isogenic diploids with low levels of DNA polymerases α and δ (reviewed by (12)). These distributions are different from that found in a wild-type strain (10).

The *pol32* diploid contained regions in which different donor chromosomes produced 4:0 conversions within a single pair of sectors (for example, Class E5, Dataset S2). This type of event occurred in 6 of 40 conversions in *pol32* compared with 1 of 140 in the wild type (Fisher’s exact test, p < 0.01) (10). We also examined the number of transitions between heterozygous regions and homozygous regions or between two homozygous regions derived from different donors in sectored colonies (Dataset S1). The transition numbers differed significantly between wild-type (10) and *pol32* strains (Fisher’s exact test, p < 0.0001), with *pol32* sectors exhibiting enrichment for both single-transition events (p = 0.002) and events containing six or more transitions (p = 0.008).

**Analysis of selected recombination events at the hotspot for recombination on chromosome VII.**

As discussed in the main text, we observed a significant clustering of recombination events in the *po32* strain located near the right telomere of chromosome VII. To confirm this observation, we constructed derivatives of the *pol32* strain (QL92) and the wild-type strain (QL90) with additional markers allowing the selection of recombination events on VII. In these strains, the *URA3* gene was inserted centromere-distal to the hotspot, and the *HIS3* gene was inserted centromere-proximal (Fig. S2). A crossover or BIR event initiated between these insertions would result in a 5-FOA^R^/Ura^-^ His^+^ derivative (Fig. S2). We measured the rates of 5-FOA^R^ and 5-FOA^R^ His^+^ derivatives by fluctuation analysis as described previously (13). In brief, we measured the frequency of FOA^R^ and 5-FOA^R^ His^+^ isolates in each of 30 independent cultures of the wild-type and *pol32* strains. These frequencies were converted to rates using the method of the median (13).

**Calculation of rates of genomic alterations**

As in previous mutation-accumulation experiments, we calculated rates of most genomic alterations per cell division by dividing the total number of events by the product of the number of times that isolates had been sub-cultured (38 isolates sub-cultured 8 times; 8 isolates sub-cultured 20 times for a total of 464 cycles), and the number of cell divisions required to form a colony. We previously determined that the colonies formed by wild-type diploids on rich medium in two days were the result of about 25 cell divisions. Based on the numbers of cells per colony in the wild-type and the *pol32* strains, we found that the colonies formed by *pol32* strains in the same amount of time had three times fewer cells, requiring an average of 23.4 doublings to form a colony. Thus, the rates of each class of event per cell division were calculated by dividing the number of events by 10858 for the *pol32* strains. Since the numbers of cells/colony were not significantly different for the wild-type and *rev3* strains or for the *pol32* and *pol32 rev3* strains, we used 25 cell doublings per cycle of sub-cloning for *rev3* and 23.4 doublings for the *pol32 rev3* in our rate calculations. We sub-cultured 16 *rev3* isolates (WY75) 60 times, the number of events were divided by 24000 in calculation of rates. For the *pol32 rev3* strain QL60, the number used to calculate rates was 9126 (13 isolates sub-cultured 30 times with 23.4 doublings per sub-culture cycle).

**Supplemental Figures**

**Figure S1. Detection of DSBs in wild-type and *pol32* strains using a fluorescently-tagged Rad52 protein.**

A. Pictures of wild-type and *pol32* strains containing Rad52 foci (shown with arrows) indicating the presence of DSBs.

B. The proportions of cells with Rad52 foci in wild-type and *pol32* cells.

**Figure S2. Analysis of chromosome VII hotspot.** The hotspot observed in *pol32* strains maps between coordinates 1000 kb and 1065 kb near the end of chromosome VII. We constructed a *pol32* strain that was heterozygous for insertions of *HIS3* and *URA3* flanking the hotspot, these insertions were located on the YJM789-derived homolog.

A. Expected pattern of LOH for crossovers between *HIS3* and *URA3*. Red and blue lines represent the chromatids derived from W303 and YJM789, respectively. A crossover between *HIS3* and *URA3* would result in one daughter cell (enclosed in a rectangle) that lost the *URA3* insertion and was, therefore, resistant to 5-fluoroorotate acid (5-FOA^R^), and retained the *HIS3* insertion (His^+^).

B. Pattern of LOH for crossovers located between *CEN7* and *HIS3*. A crossover between *CEN7* and *HIS3* would result in one daughter cell (enclosed in a rectangle) that lost both the *HIS3* and *URA3* insertions (5-FOA^R^ and His^-^ phenotype).

C. Bar graph showing the rate of recombination at the hotspot. This graph shows the rates of recombination between *CEN7* and *URA3* (left side of figure), and *HIS3* and *URA3* (right side of figure) for both wild-type and *pol32* strains. 95% confidence limits are shown for all rates.

**Figure S3. Patterns of LOH at the chromosome VII hotspot in *pol32* isolates (DNA sequence analysis).** Fourteen independent isolates with the 5-FOA^R^ His^+^ genotype (see Fig. S2) were examined by whole-genome sequencing. Red and blue lines indicate "relative coverage" (RC) for W303- and YJM789-associated SNPs, respectively. RC values of 0.5 indicate heterozygous SNPs and values of 1.0 and 0 signify LOH. Multiple LOH transitions are obvious in most of the *pol32* isolates.

**Figure S4. Patterns of LOH at the chromosome VII hotspot in wild-type isolates (microarray analysis).** As in Fig. S3, red and blue indicate SNPs derived from W303 and YJM789, respectively. "Hybridization ratio" (HR) values of 1 indicate heterozygous SNPs and values of 1.5 and 0.5 signify LOH. Twelve isolates were examined. Most isolates had a single LOH transition with only two exceptions

**Figure S5. I-DEL event in isolate WYpol32-8-7 resulting from non-allelic recombination events that produce two translocations.**

A. Short-read sequence analysis of WYpol32-8-7. From this analysis, we detected a 50 kb I-DEL event on chromosome V, but no LOH event on chromosome XIII.

B. Long-read sequence analysis of WYpol32-8-7. By Nanopore sequencing, we detected reads that included a translocation between chromosomes V and XIII with a breakpoint at coordinate 442 kb on V and 372 kb on XIII. In addition, there was a second translocation with a breakpoint as coordinate 372 kb on XIII and 492 kb on V. There were Ty elements located as each of these breakpoints.

C. Mechanism to explain the translocations in WYpol32-8-7. In this figure, chromosome V and XIII sequences are shown in blue and brown, respectively. The W303- and YJM789-derived homologs are shown as undotted and dotted lines respectively. We suggest that the event was initiated by a G1-associated DSB in a Ty element located on chromosome XIII. Following DNA replication, there would be two W303-derived chromatids with DSBs at the same Ty element. We suggest that one of the breaks was repaired using a Ty element at position 442 kb on V and the second repaired using a Ty element at position 492 kb on a sister chromatid. After segregation as indicated by arrows in the figure, one daughter cell (shown in a rectangle) would contain the 50-kb deletion of chromosome V sequences without a change in the number of sequences on chromosome XIII.

**Figure S6. Effect of the *pol32* mutation on the number of copies of ribosomal DNA (rDNA) and *CUP1* sequences, telomere length, and chromosome disjunction.**

A. Number of rDNA repeats in wild-type and *pol32* diploids. The rDNA genes are located in a single tandem array on chromosome XII. We determined the number of rDNA repeats/cell by determining sequence coverage of the rDNA relative to single-copy genes. Since the rDNA of W303 and YJM789 have SNPs that distinguish them, we could also monitor copy-number for each class of repeat separately. The total numbers of repeats as well as the numbers of repeats of each type are reduced in *pol32* strains compared to the isogenic wild-type strain. 95% confidence limits are shown by brackets. The median rDNA copy number was 186 in wild-type diploid isolates (95% confidence limits [CL], 182–194) and 134 in WYpol32-derived isolates (95% CL, 119–155).

B. Number of *CUP1* repeats in wild-type and *pol32* strains. Using methods similar to those employed for the rDNA, we showed that the *pol32* strains had significantly smaller numbers of *CUP1* repeats. The median copy number of the *CUP1* repeats was reduced, from 16 (95 % CL 15-17) in the wild-type strain to 12 (95 % CL 10-14) in the *pol32* strain; the values are significantly different (p < 0.001 by Mann-Whitney tests).

C. Length of telomeres in wild-type and *pol32* strains. Yeast telomeric sequences have the form poly G_1-3_T. By long-read sequencing, we measured the average lengths of these tracts in wild-type and *pol32* strains. Tract lengths in *pol32* strains were significantly larger.

D. Patterns of aneuploidy in sub-cultured diploid *pol32* isolates. This figure summarizes the data from Dataset S1-5. The abbreviations are: MONO (monosomic), EUP (euploid), TRI (trisomic), UPD (uniparental disomy), and TETRA (tetrasomic). Each WYpol32 isolate was sub-cultured separately.

**Figure S7. Patterns of mitotic recombination detected by analyzing samples derived from sectored colonies.** The red and blue colors indicate the homolog was derived from W303 and YJM789, respectively; ovals/circles represent centromeres. *SUP4-o* encodes an ochre-suppressor tRNA. The diploid WYpol32 is homozygous for both *pol32* and the ochre-suppressible *ade2-1* allele. When unsuppressed, the *ade2-1* mutation results in a red colony, while form pink or white colonies, respectively, with one or two copies of *SUP4-o*. A reciprocal crossover produces a red/white sectored colony.

A. Reciprocal T-LOH event associated with a gene conversion event resulting from mitotic recombination initiated in S/G2. The region of gene conversion is detected by a difference in LOH breakpoints for the two sectors. Within the boxed region, three of the chromosomes contain YJM789-specific SNPs and one has a W303-specific SNP. This pattern of 3:1 conversion is consistent with an event initiated after chromosome replication on one chromatid.

B. Reciprocal T-LOH event associated with a 4:0 gene conversion event resulting from reciprocal mitotic recombination initiated in G1. In this event, a DSB in an unreplicated chromosome is formed. Replication of the broken chromosome results in two sister chromatids broken at the same position. Repair of one of the DSBs results in a reciprocal crossover whereas the other DSB is repaired without a crossover.

C. Reciprocal T-LOH event associated with a 4:0/3:1 hybrid gene conversion event resulting from mitotic recombination initiated in G1.

D. Example of a red/white sector: reciprocal crossover associated with a 3:1 conversion event diagnosed by microarrays. Heterozygous regions have a normalized hybridization ratio (HR) value of about 1, regions homozygous for the SNP have an HR value of about 0.5, and regions in which the SNP have a HR of about 0.2 (10). In the top two panels, we show the analysis of the white sector with low (left side) and high (right side) resolution. The arrow shows the region of the LOH transition. In the bottom two panels, we show the analysis of the red sector.

**Figure S8. Mechanisms that could produce multiple switches of LOH regions such as those observed in the hotspot for LOH events on the right arm of chromosome VII.** Thick red and blue lines represent chromosomes with the different colors showing the two homologs. The strain is heterozygous for a *URA3* insertion near the end of the chromosome. Ovals and circles indicate centromeres and the horizontal X shows a crossover.

A. Switching of LOH regions as the result of symmetric heteroduplex formation. A G1-initiated on the red homolog results in two broken red chromatids. Repair of these chromatids results in very long heteroduplex regions as the result of branch migration of Holliday junctions located at the break. The heteroduplexes are indicated by thin red and blue lines. The heteroduplex intermediate between chromatids 2 and 3 is associated with a crossover whereas the heteroduplex involving 1 and 4 is not. The patchy mismatch repair of the heteroduplex results in regions of LOH that have different donors. Segregation of chromatids 2 and 4 yield a daughter cell that lacks the *URA3* insertion and is, therefore, resistant to 5-fluoro-orotate.

B. Adjacent LOH regions with different donors as a consequence of multiple DSBs on both homologs. Perhaps as a consequence of delayed replication, the chromatids have multiple DNA breaks. During repair of those DSBs, both the red and blue chromosomes are used as donors of information. The net results of these repair processes are cells that have LOH regions that duplicate blue sequences and delete red sequences next to regions that duplicate red sequences and delete blue.

**Figure S9. Location of breakpoints for reciprocal crossovers on chromosome IV in wild-type and *pol32* strains.** Formation of red/white sectors require that the crossover occur between *CEN4* (SGD coordinate 450 kb) and the heterozygous *SUP4-o* gene (coordinate 1510 kb). We show the number of "windows" that include the breakpoints for I-LOH and T-LOH events in sectored colonies for the wild-type strain and the isogenic *pol32* strain. The windows were determined as described in the legend to Fig. 2.

A. Mapping of wild-type LOH breakpoints (10).

B. Mapping of LOH breakpoints in *pol32* strain.

C. Overlap of wild-type and *pol32* breakpoints.

**Table S1. Strain constructions and strain list**

| **Name** | **Background** | ***Genotype*** | **Construction** |
| --- | --- | --- | --- |
| JSC25 | W303×YJM789 | *MAT***a***/MATα::HYG LEU2/leu2-3,112 HIS3/his3-1115 ura3/ura3-1 GAL2/gal2 ade2-1/ade2-1 TRP1/trp101 CAN1/can1-100:: NatMX4 RAD5/RAD5 IV1510386:: KanMX6 -can1-100/IVI1510386::SUP4-o* | (10) |
| WYspo11 | W303×YJM789 | *MAT***a***/α leu2-3,112/LEU his3-11,15/HIS ura3-1/ura3 ade2- 1/ade2-1 trp1- 1/TRP can1-100/CAN1 RAD5/RAD5 GAL2/gal2 ho::hisG IV1510386/IV1510386::SUP4-o spo11::loxP-KanMX6-loxP/spo11::loxP-KanMX6-loxP* | (14) |
| Wspo11 | W303 | *MAT***a** *leu2-3,112 his3-11,15 ura3-1 ade2-1 trp1-1 can1-100 RAD5 spo11::loxP-KanMX6-loxP* | (14) |
| Yspo11 | YJM789 | *MATα ade2-1 ura3 gal2 ho::hisG IV1510386::SUP4-o spo11::loxP-KanMX6-loxP* | (14) |
| Wpol32 | W303 | *MAT***a** *leu2-3,112 his3-11,15 ura3-1 ade2-1 trp1-1 can1-100 spo11::loxP-KanMX6-loxP pol32::URA3* | *POL32* was deleted in Wspo11 using a *URA3* cassette amplified from pUG72 with primers dPOL32S and dPOL32A. |
| Ypol32 | YJM789 | *MATα ade2-1 ura3 gal2 ho::hisG IV1510386::SUP4-o spo11::loxP-KanMX6-loxP pol32::URA3* | *POL32* deleted in Yspo11 as described above for Wpol32. |
| WYpol32 | W303×YJM789 | *MAT***a***/α leu2-3,112/LEU2 his3-11,15/HIS3 ura3-1/ura3 ade2-1/ade2-1 trp1-1/TRP1 can1-100/CAN1 GAL2/gal2 ho::hisG/ho IV1510386/IV1510386::SUP4-o spo11::loxP-KanMX6-loxP/spo11::loxP-KanMX6-loxP pol32::URA3/pol32::URA3* | Mating of Wpol32 with Ypol32. For sub-cultured WYpol32 strains, we used the following nomenclature: a. WYpol32-8-1 to WYpol32-8-59 indicates individual isolates sub-cultured (single cell to a colony) 8 times at 30 °C. The last number indicates the individual isolates in Dataset S1-1.  b. WYpol32-20-32 to WYpol32-20-46 indicates isolates sub-cultured 20 times at 30 °C (see Dataset S1-1). |
| Wspo11-loxP | W303 | *MAT***a** *leu2-3,112 his3-11,15 ura3-1 ade2-1 trp1-1 can1-100 RAD5 spo11::loxP* | Transformed Wspo11 with the Cre-expressing plasmid pSH65 (15) to delete *KanMX6.* Obtained a strain that subsequently lost pSH65. |
| Yspo11-loxP | YJM789 | *MATα ade2-1 ura3 gal2 ho::hisG IV1510386::SUP4-o spo11::loxP* | Deleted *KanMX6* as described for Wspo11-loxP |
| Wrev3 | W303 | *MAT***a** *leu2-3,112 his3-11,15 ura3-1 ade2-1 trp1-1 can1-100 RAD5 spo11::loxP rev3::NatMX4* | Used a cassette containing *NatMX4* (obtained by amplifying pAG25 (16) with primers dREV3S and dREV3A) to delete *REV3*. |
| Yrev3 | YJM789 | *MATα ade2-1 ura3 gal2 ho::hisG IV1510386::SUP4-o spo11::loxP rev3::NatMX4* | Used same method describe for construction of Wrev3 to delete *REV3*. |
| WY75 | W303×YJM789 | *MAT***a***/α leu2-3,112/LEU2 his3-11,15/HIS3 ura3-1/ura3 ade2-1/ade2-1 trp1-1/TRP1 can1-100/CAN1 GAL2/gal2 ho::hisG IV1510386/IV1510386::SUP4-o spo11::loxp/spo11::loxp rev3::NatMX4/rev3::NatMX4* | Mating of Wrev3 and Yrev3. Sub-cultured derivatives (60 cycles at 30°C) were labeled WY75-60-2 to WY75-60-20. |
| QL58 | W303 | *MAT***a** *leu2-3,112 his3-11,15 ura3-1 ade2-1 trp1-1 can1-100 spo11::loxp rev3::NatMX4 pol32::URA3* | *POL32* was deleted in the W rev3 strain using a *URA3* cassette obtained by amplifying pUG72 (15) with primers dPOL32S and dPOL32A. |
| QL59 | YJM789 | *MATα ade2-1 ura3 gal2 ho::hisG can1::NAT IV1510386::SUP4-o spo11::loxp rev3::NATMX4 pol32::URA3* | *POL32* was deleted in the Yrev3 strain using the same approach described for QL58. |
| QL60 | W303×YJM789 | *MAT***a***/α leu2-3,112/LEU2 his3-11,15/HIS3 ura3-1/ura3 ade2-1/ade2-1 trp1-1/TRP1 can1-100/CAN1 GAL2/gal2 ho::hisG IV1510386/IV1510386::SUP4-o spo11::loxp/spo11::loxp rev3::NatMX4/rev3::NatMX4 pol32::URA3/pol32::URA3* | Cross of QL58 with QL59. Individual isolates of QL60 were sub-cultured 30 times at 30°C. Individual sub-cultured isolates were designated: QL60-30-1 to QL60-30-29. |
| YZ26 | W303 | *MATα leu2-3,112 his3-11,15 ura3-1 ade2-1 trp1-1 can1-100::NatMX4* | (1) |
| YJM842 | YJM789 | *MAT***a** *ho::hisG his3Δ-200 ura3* | (13) |
| QL71 | W303 | *MATα leu2-3,112 his3-11,15 ura3-1 ade2-1 trp1-1 can1-100::NatMX4 spo11::KanMX6* | To delete *SPO11*, we used a PCR fragment containing the *KanMX6* cassette obtained by amplifying genomic DNA from Wspo11 with primers pspo11S and pspo11A to transform YZ26. |
| QL81 | W303 | *MATα leu2-3,112 his3-11,15 ura3-1 ade2-1 trp1-1 can1-100::NatMX4 spo11::KanMX6 pol32::* *HphMX4* | The *POL32* gene was deleted from QL71 using a *HphMX4*-containing cassette resulting from PCR amplification of pAG32 (16) with primers dPOL32S and dPOL32A. |
| QL72 | YJM789 | *MAT***a** *ho::hisG his3Δ-200 ura3 spo11::KanMX6* | The *SPO11* gene was deleted from YJM842 using the same procedure used in the construction of QL71. |
| QL74 | YJM789 | *MAT***a** *ho::hisG his3Δ-200 ura3 spo11::KanMX6 VII1065736::URA3* | *URA3* was inserted at position 1065736 on chromosome VII in the QL72 strain. The *URA3* cassette was amplified from pUG72 (16) using primers VIIR-URA3S and VIIR-URA3A. |
| QL78 | YJM789 | *MAT***a** *ho::hisG his3Δ-200 ura3 spo11::KanMX6 VII1065736::URA3 VII1000193::HIS3* | *HIS3* was inserted at 1065736 on chromosome VII in QL74. The *HIS3* cassette was amplified from genomic DNA of S288C, with the primers VIIR-HIS3S and VIIR-HIS3A |
| QL80 | YJM789 | *MAT***a** *ho::hisG his3Δ-200 ura3 spo11::KanMX6 VII1065736::URA3 VII1000193::HIS3 pol32::NatMX4* | *POL32* in QL78 was deleted using the *NatMX4* cassette generated by amplifying pAG25 (16) with primers pPOL32S and pPOL32A. |
| QL90 | W303×YJM789 | *MAT***a***/MATα LEU2/leu2-3,112 his3Δ-200/his3-11,15 ura3/ura3-1 ADE2/ade2-1 TRP1/trp1-1* *CAN1/can1-100::NATMX4 VII1000193/VII1000193::HIS3 VII1065736/VII1065736::URA3 spo11::KanMX6/spo11::KanMX6* | Cross of QL71 with QL78. |
| QL92 | W303×YJM789 | *MAT***a***/MATα LEU2/leu2-3,112 his3Δ-200/his3-11,15 ura3/ura3-1 ADE2/ade2-1 TRP1/trp1-1* *CAN1/can1-100::NatMX4 spo11::KanMX6/spo11::KanMX6 VII1065736/VII1065736::URA3 VII1000193/VII1000193::HIS3 pol32::HphMX4/pol32::NatMX4* | Cross of QL80 with QL81. |
| X8068-6C | W303 | *MAT***a** *can1-100 ura3-1 his3-11,15 leu2-3,112 trp1-1 RAD52-YFP* | (17) |
| X8068-6Cpol32 | W303 | *MAT***a** *can1-100 ura3-1 his3-11,15 leu2-3,112 trp1-1 RAD52-YFP pol32::URA3* | *pol32* deletion generated by transformation of X8068-6C with PCR fragment obtained by amplifying pUG72 plasmid DNA with primers dPOL32S and dPOL32A. |

**Table S2 Primers used in this study.**

| **Primer** | **Sequence (5' to 3')** | **Purpose** |
| --- | --- | --- |
| dPOL32S | GCTCGAAATAATATTTCACATTAACTAACAACCAGAAATAGGCTGCAGGTCGACAACCC | Delete *POL32* using pUG72/pAG25 as the PCR template. |
| dPOL32A | TCACGTAAGTTGACATTTGTATTATACATTACATCACAATTAGTGGATCTGATATCACC |  |
| Vpol32A | TGAAACCATTATTGAAGCAG | Verify the deletion of *POL32* |
| vHygS | GAGGGCAAAGGAATAATCAG |  |
| vNATS | CTGCCCAGATGCGAAGTT |  |
| vpug72S | TATGATTGTCTCCGTAAGCT |  |
| vpUG6S | CTTCATTACAGAAACGGCT |  |
| pspo11S | GGGTAAACATTCATCCATTG | Amplify the *spo11::KanMX6* cassette from Wspo11 genomic DNA |
| pspo11A | GGTTGCTATTTCGATTCTG |  |
| vspo11A | GGTTCGATTCTAGGAGATG | Verify the deletion of *SPO11*, using vpUG6S as upstream primer |
| dREV3S | TACAAAACTACAAGTTGTGGCGAAATAAAA TGTTTGGAAATGTCGTACGCTGCAGGTCGA | Delete *REV3* using pAG25 as the PCR template. |
| dREV3A | TACTCATCATTTTGCGAGACATATCTGTGT CTAGATTACCAATCATCGATGAATTCGAGC |  |
| vREV3S | AGGGCAAAGGAATAATCAG | Verify the deletion of *REV3* |
| vREV3A | TCCTTCTCCGAAATAGTACC |  |
| VIIR-URA3S | GTTTCGTTGGAGATGCAGTAATAATGCAGAATACGGATCTCACGGACCCCAAGAGATCCCAATACAACAG | Insert *URA3* on chromosome VII at coordinate 1065736 |
| VIIR-URA3A | CATTTTGCATCGATTGGGAACTGAGAACCTACAAGCCGTAAGATTGCGTAGCTTTAGGTTCTATCGAGGA |  |
| vVIIR-URA3A | GACCTAGTTATTCATTGCGA | Verify the insertion of *URA3* on chromosome VII, using vpug72S as the upstream primer. |
| VIIR-HIS3S | TGCCGCGGGCACTTGAGCACCTCATGCACAGCAATAACACAACACAATGGTTAGTGCACCATAAATTCCC | Insert *HIS3* on chromosome VII at coordinate 1000193 |
| VIIR-HIS3A | GACTTTTCTGGTTGTCCCGCTTCACGGCACATGCATGCATCAATGACCGAATTCTCTTACGCATCTGTGC |  |
| vHIS3S | ACCGTAGTGAGAGTGCGTTC | Verify the insertion of *HIS3* on chromosome VII |
| vVIIR-HIS3A | TGCGACAATTTGTGATATGC |  |

**Table S3. Numbers and rates of genome alterations in *pol32, rev3,* and *pol32 rev3* diploids.**

| **Genome alteration^1^** | **Rate**×**10^-3^/cell division in *pol32*^2^** | **Number of events in *pol32 rev3*** | **Rate**×**10^-3^/cell division in *pol32 rev3*^3^** | **Ratio of *pol32 rev3* rate to *pol32* rate** | **Ratio of *pol32 rev3* rate to wild-type rate^4^** | **Number of events in *rev3*** | **Rate** ×**10^-3^/cell division in *rev3*^5^** | **Ratio of WT rate to *rev3* rate** |  |
| --- | --- | --- | --- | --- | --- | --- | --- | --- | --- |
| I-LOH | 15  (12-17) | 39 | 4.3  (3.0-5.8)* | 0.29 | 1.3 | 94 | 3.9  (3.2-4.8) | 0.85 |  |
| T-LOH | 18  (15-20) | 55 | 6.0  (4.5-7.9) * | 0.33 | 4.6* | 42 | 1.8  (1.3-2.4) | 0.72 |  |
| I-DEL | 2.1  (1.3-3.2) | 5 | 0.55  (0.18-1.3) * | 0.26 | 5.5* | 0 | 0 | ND |  |
| I-DUP | 0.28  (0.06-0.81) | 4 | 0.44  (0.12-1.1) | 1.6 | 15* | 1 | 0.04  (0.001-0.23) | 0.75 |  |
| T-DEL | 0.83  (0.38-1.6) | 3 | 0.33  (0.07-0.96) | 0.40 | 17* | 0 | 0 | ND |  |
| T-DUP | 0.74  (0.32-1.5) | 2 | 0.22  (0.03-0.79) | 0.30 | 22* | 0 | 0 | ND |  |
| Aneuploidy and UPD | 3.0  (2.0-4.2) | 18 | 2.0  (1.2-3.1) | 0.67 | 33* | 1 | 0.04  (0.001-0.23) | 1.5 |  |
| Single base mutations | 8.2  (6.6-10) | 50 | 5.5  (4.1-7.2)* | 0.67 | 1.1 | 83 | 3.5  (2.8-4.3) * | 1.5 |  |
| Small in/dels and complex mutations | 1.3  (0.71-2.2) | 7 | 0.71  (0.31-1.6) | 0.55 | 2.6* | 6 | 0.25  (0.09-0.54) | 1.08 |  |

^1^The various types of genomic alterations are described in the legend to Table 1 and listed in Dataset S3.

^2^The data for the *pol32* strain were derived from Table 1.

^3^Rates were calculated by dividing the number of events by 9126 (the number of cell divisions of isolate during sub-culturing × the number of isolates). Rates are expressed as ×10^-3^ per cell division. Values in brackets indicate 95% confidence limits. Asterisks indicate that the *pol32 rev3* rate was not within the 95% confidence limits of the *pol32* rate.

^4^ Asterisks indicate that the wild-type rate was not within the 95% confidence limits of the *pol32 rev3* rate.

^5^Rates were calculated by dividing the number of events by 24000 (the number of cell divisions of isolate during sub-culturing × the number of isolates). Asterisks indicate that the wild-type rate (Table 1) was not within the 95% confidence limits of the *rev3* rate. Only the rates of single-base mutations were significantly different by this criterion.

**Table S4. Ratio of LOH events that are I-LOH and T-LOH for strains of different genotypes.**

| **Genotype^1^** | **Number of I-LOH events** | **% of I-LOH events^2^** | **Number of T-LOH events** | **% of T-LOH events^2^** | **p value compared to wild-type^3^** |  |
| --- | --- | --- | --- | --- | --- | --- |
| Wild-type | 859 | 71% | 356 | 29% | 1 |  |
| *pol32* | 158 | 45% | 193 | 55% | <0.0001 |  |
| *pol32 rev3* | 39 | 42% | 55 | 58% | <0.0001 |  |
| *rev3* | 94 | 69% | 42 | 31% | 0.77 |  |
| Low Pol α | 86 | 43% | 115 | 57% | <0.0001 |  |
| Low Pol δ | 21 | 23% | 69 | 77% | <0.0001 |  |
| Low Pol ε | 27 | 23% | 89 | 77% | <0.0001 |  |

^1^The numbers of I-LOH and T-LOH events for the *pol32*, *rev3*, and *pol32 rev3* strains are given in this paper. The references for the other data are: Low Polα (18), Low Pol δ (13), Low Pole ε (1). In the strains with low levels of various DNA polymerases, the levels were controlled using fusions of the DNA polymerase genes to a galactose-inducible promoter. The levels of DNA polymerases in these strains in medium with low levels of galactose were approximately 10% of the wild-type levels.

^2^The percentages were calculated by dividing the number of events in each category by the total number of LOH events (I-LOH plus T-LOH).

^3^The statistical significance of the comparisons were calculated by comparing the numbers of events in each class in the various mutant strains to the numbers of events in the wild-type strain using the Chi-square test.

**Supplemental References**

1. Zhang K, Sui Y, Li WL, Chen G, Wu XC, Kokoska RJ, Petes TD, Zheng DQ. 2022. Global genomic instability caused by reduced expression of DNA polymerase ε in yeast. Proc Natl Acad Sci U S A 119:e2119588119.

2. Li H, Durbin R. 2009. Fast and accurate short read alignment with Burrows-Wheeler transform. Bioinformatics 25:1754-60.

3. Li H, Handsaker B, Wysoker A, Fennell T, Ruan J, Homer N, Marth G, Abecasis G, Durbin R, Genome Project Data Processing S. 2009. The Sequence Alignment/Map format and SAMtools. Bioinformatics 25:2078-9.

4. Koboldt DC, Zhang Q, Larson DE, Shen D, McLellan MD, Lin L, Miller CA, Mardis ER, Ding L, Wilson RK. 2012. VarScan 2: somatic mutation and copy number alteration discovery in cancer by exome sequencing. Genome Res 22:568-76.

5. Qi L, Sui Y, Tang XX, McGinty RJ, Liang XZ, Dominska M, Zhang K, Mirkin SM, Zheng DQ, Petes TD. 2023. Shuffling the yeast genome using CRISPR/Cas9-generated DSBs that target the transposable Ty1 elements. PLoS Genet 19:e1010590.

6. McGinty RJ, Rubinstein RG, Neil AJ, Dominska M, Kiktev D, Petes TD, Mirkin SM. 2017. Nanopore sequencing of complex genomic rearrangements in yeast reveals mechanisms of repeat-mediated double-strand break repair. Genome Res 27:2072-2082.

7. Sedlazeck FJ, Rescheneder P, Smolka M, Fang H, Nattestad M, von Haeseler A, Schatz MC. 2018. Accurate detection of complex structural variations using single-molecule sequencing. Nat Methods 15:461-468.

8. St Charles J, Hazkani-Covo E, Yin Y, Andersen SL, Dietrich FS, Greenwell PW, Malc E, Mieczkowski P, Petes TD. 2012. High-resolution genome-wide analysis of irradiated (UV and gamma-rays) diploid yeast cells reveals a high frequency of genomic loss of heterozygosity (LOH) events. Genetics 190:1267-1284.

9. Barbera MA, Petes TD. 2006. Selection and analysis of spontaneous reciprocal mitotic crossovers in *Saccharomyces cerevisiae*. Proc Natl Acad Sci U S A 103:12819-12824.

10. St Charles J, Petes TD. 2013. High-resolution mapping of spontaneous mitotic recombination hotspots on the 1.1 Mb arm of yeast chromosome IV. PLoS Genet 9:e1003434.

11. Symington LS, Rothstein R, Lisby M. 2014. Mechanisms and regulation of mitotic recombination in *Saccharomyces cerevisiae*. Genetics 198:795-835.

12. Zheng DQ, Petes TD. 2018. Genome instability induced by low levels of replicative DNA polymerases in yeast. Genes 9:539-559.

13. Zheng DQ, Zhang K, Wu XC, Mieczkowski PA, Petes TD. 2016. Global analysis of genomic instability caused by DNA replication stress in *Saccharomyces cerevisiae*. Proc Natl Acad Sci U S A 113:E8114-8121.

14. Sui Y, Qi L, Wu JK, Wen XP, Tang XX, Ma ZJ, Wu XC, Zhang K, Kokoska RJ, Zheng DQ, Petes TD. 2020. Genome-wide mapping of spontaneous genetic alterations in diploid yeast cells. Proc Natl Acad Sci U S A 117:28191-28200.

15. Gueldener U, Heinisch J, Koehler GJ, Voss D, Hegemann JH. 2002. A second set of marker cassettes for Cre-mediated multiple gene knockouts in budding yeast. Nucleic Acids Research 30.

16. Goldstein AL, McCusker JH. 1999. Three new dominant drug resistance cassettes for gene disruption in *Saccharomyces cerevisiae*. Yeast 15:1541-1553.

17. Meng XZ, Wei L, Peng XP, Zhao XL. 2019. Sumoylation of the DNA polymerase ε by the Smc5/6 complex contributes to DNA replication. PLoS Genet 15.

18. Song W, Dominska M, Greenwell PW, Petes TD. 2014. Genome-wide high-resolution mapping of chromosome fragile sites in *Saccharomyces cerevisiae*. Proc Natl Acad Sci U S A 111:E2210-2218.
